# Experiences of Postpartum Follow-Up and Participation in a Lifestyle Intervention after Gestational Diabetes: A Qualitative Study

**DOI:** 10.3390/nu16203487

**Published:** 2024-10-15

**Authors:** Siri Ressem Gustavsen, Astrid Vatn Wensbakk, Heidi Linn Sandsæter, Julie Horn

**Affiliations:** 1 Department of Public Health and Nursing, Norwegian University of Science and Technology, 7030 Trondheim, Norway; sirirg@stud.ntnu.no (S.R.G.); astrivw@stud.ntnu.no (A.V.W.); heidi.l.sandsater@ntnu.no (H.L.S.); 2 Department of Obstetrics and Gynecology, Levanger Hospital, Nord-Trøndelag Hospital Trust, 7600 Levanger, Norway

**Keywords:** gestational diabetes mellitus, health promotion, intervention, lifestyle changes, postpartum, prevention, women’s health

## Abstract

Background/Objectives: Gestational diabetes is associated with an increased risk of future type 2 diabetes and cardiovascular disease, but healthy lifestyle changes can prevent the development of these diseases. This study aimed to identify factors that can improve intervention programmes and postpartum support after gestational diabetes. Methods: Twenty-two women who had experienced gestational diabetes in Norway participated in in-depth interviews following a six-month intervention programme focusing on healthy lifestyle changes. Participants were included 3–12 months after giving birth. The data were analysed using reflexive thematic analysis. Results: Four themes were developed: (1) A status report on my health and lifestyle—crucial for recognising the need for change; (2) encouragement and cheering on: getting started and maintaining changes; (3) life’s challenging moments: looking after the baby and prioritising one’s own health; and (4) the first period with the newborn baby—a good time to make changes. Participants described maternity leave as a suitable time for lifestyle change. Adequate information about and insight into their health were important for success. The focus on small changes motivated them to improve their lifestyle. Participants emphasised individualised help, support from others, noticing an improvement and seeing a positive effect on their family members as motivational factors for maintaining the changes. However, they found it difficult to prioritise themselves and to maintain lifestyle changes in challenging life situations and transitional phases. Conclusions: The study findings can help support the development of future intervention programmes for women who have experienced gestational diabetes.

## 1. Introduction

Gestational diabetes mellitus (GDM) is a condition of hyperglycaemia that occurs during pregnancy [1]. The condition usually disappears soon after birth, but women who have had GDM have an eightfold times higher risk of developing type 2 diabetes mellitus (T2DM) [2] and twice the risk of future cardiovascular disease (CVD) [3], compared to women with normal glucose levels in pregnancy.

The prevalence of GDM among pregnant women varies with ethnicity, the diagnostic criteria used and the amount of screening [4]. In 2022, the prevalence of GDM was 6.5% in pregnant women in Norway [5]. In recent decades, prevalence has increased both nationally and internationally. This increase is thought to be due to higher age and a greater prevalence of obesity among pregnant women [1,6].

Lifestyle changes have been shown to reduce the development of T2DM in women who have had GDM by up to 50% [7], and the postpartum period has been identified as the ideal time for lifestyle modification [8]. As in many other countries, clinical guidelines in Norway recommend giving women who have had GDM advice and practical support for lifestyle changes in the postpartum period [1,9].

However, there is variation in postpartum support of women with GDM and many women have called for more help from healthcare providers to enable them to make lifestyle changes [10,11]. Although women with GDM are often motivated to change their lifestyle postpartum, many find it difficult [12,13].

A number of barriers to lifestyle change have been identified in this patient group, including limited knowledge, social support, time, and energy [13]. Intervention programmes designed to address the unique challenges experienced by women with GDM in the postpartum period have the potential to facilitate healthy lifestyle changes in the crucial phase of early parenthood, with an important additional effect on the women’s family members.

This study explores women’s experiences of postpartum follow-up and participation in “Mom’s Healthy Heart” (MHH), a lifestyle intervention programme for women after a pregnancy complicated by GDM. The aim of the study is to identify factors that can help to design and improve future intervention programmes and enhance understanding of how postpartum support for this patient population can be optimised.

## 2. Materials and Methods

A qualitative research design was used to explore the participants’ experiences of postpartum follow-up and participation in a lifestyle intervention programme after a pregnancy with GDM.

### 2.1. Study Design

This study is based on data from the pilot intervention study “Mom’s Healthy Heart”. The study participants in MHH were women in Trøndelag County in central Norway with recent pre-eclampsia or GDM. The aim of the pilot study was to explore the feasibility and effects of a lifestyle intervention for women 3–12 months postpartum [14]. The intervention focused on increasing physical activity and adherence to the national dietary guidelines [15].

During the six-month intervention programme, the study participants were given access to digital learning material on diet and physical activity, as well as telephone guidance sessions with a clinical nutritionist. At the start of the programme, and after three and six months, the participants completed a questionnaire. On the same occasions, blood samples were taken and blood pressure, body composition, and physical activity were measured. On the last occasion, the participants were invited to a semi-structured telephone interview.

### 2.2. Recruitment and Participants

Women over the age of 18 who had been diagnosed with pre-eclampsia and/or GDM and had given birth at two Norwegian hospitals were invited to participate in the MHH study. Diagnoses of pre-eclampsia and GDM were confirmed by medical record review. The recruitment period was from February to September 2020. Potentially eligible patients with a confirmed diagnosis of GDM were contacted by letter, and those interested in participation returned a signed consent form. Candidates were excluded if they had chronic diseases that required particular dietary recommendations. Further reasons for exclusion were living more than a two-hour drive from the study site, pregnancy, or poor knowledge of Norwegian. Forty-four women, 21% of those invited, were included in the study. Forty participants completed the intervention programme; 23 of these had GDM and 17 had pre-eclampsia. Twenty-two of the participants with GDM completed the interview after the intervention programme, and these constituted our study population.

### 2.3. Data Collection

At baseline, the participants’ socio-demographic data were collected via a questionnaire. During the interviews, a semi-structured interview guide was used to enable an open attitude to the participants’ spontaneous experiences and descriptions (Table S1). Due to the COVID-19 pandemic, and to provide flexibility for the participants, the interviews were conducted over the phone by the last author (JH). She had had no contact with the participants during the study. The interviews lasted between 18 and 39 min and were audiotaped. The interviewer explored the participants’ experiences of the intervention and their motivational factors and barriers to lifestyle change. The women’s experiences of the healthcare they received during pregnancy and postpartum were also examined.

### 2.4. Data Analysis

The analysis used an inductive approach, where themes were developed directly from the data (a ‘bottom-up’ orientation). Reflexive thematic analysis as described by Braun and Clarke was used because it is a flexible analysis method that enables interpretation of the data. Since lifestyle and increased risk of disease are complex and sensitive topics, it was important to be able to interpret meaning that was not directly expressed.

Reflective thematic analysis recommends using six phases: (1) familiarisation, (2) coding, (3) generating initial themes, (4) reviewing and developing themes, (5) refining and defining themes, and (6) producing the report (the results) [16]. The analysis process was led by SRG and AVW, but all six phases were discussed with JH and HLS. An important advantage of involving several researchers in the analysis is that the researchers challenge each other’s interpretations and views and uncover important blind spots.

In the first phase, both first authors (SRG and AVW) familiarised themselves with the data through the transcriptions of the 22 audio recordings. The participants were given pseudonyms, and personally identifying information, such as occupation and place of residence, was removed. For further familiarisation with the data, the transcripts were read repeatedly. Ideas and thoughts that emerged were noted down in reflection notes. The reflection notes were further developed and used throughout the analysis to ensure continuous reflection on the findings and interpretations.

In phase two, the data were reviewed systematically and data segments that appeared interesting or meaningful in relation to the research topic were coded. In order to elicit more possible aspects and interpretations of the data, AVW and SRG discussed the meaning of the participants’ statements during the process. The iterative coding process resulted in twelve codes. Groups of codes with a similar meaning or concept were grouped into preliminary themes. In phase four, themes were further developed and refined. Discussions between all authors led to the generation of relevant themes with several subthemes. The data associated with each theme were reviewed to ensure that the themes described patterns of meaning and that important participant experiences had not been omitted. In addition, the interrelationship between the themes and the relationship between the themes and the entire dataset and the research topic were assessed. This recursive process involved defining the boundaries between themes, merging or splitting initial themes, guided by thematic mapping (Figure S1) and discussions within the research group. In the fifth phase, further adjustments were made, including naming themes and writing theme definitions. Each theme definition clarified its content and limits to prevent overlap between themes. Lastly, the report was written in phase six. The analysis resulted in four final themes.

### 2.5. Ethics

The Central Norway Regional Committee for Medical and Health Research Ethics granted ethical approval to conduct the intervention study, including this qualitative study (reference number: 2018/1803). All study participants provided written consent to participate in the intervention and interviews. At the beginning of the interviews, verbal consent was obtained to make audio recordings of the conversations, and the participants were informed about privacy and the fact that all data would be anonymised or deleted after processing.

## 3. Results

The 22 participants varied in age, marital status, level of education, household income, body mass index, and parity. Most were born and raised in Norway, but two came from countries outside Scandinavia. The participants’ sociodemographic and pregnancy-related characteristics are presented in Table 1.

The participants mentioned a number of motivators and barriers to lifestyle change following a pregnancy with GDM. These are described in the following four themes:

1. A status report on my health and lifestyle—crucial for recognising the need for change;
2. Encouragement and cheering on: getting started and maintaining changes;
3. Life’s challenging moments: looking after the baby and prioritising one’s own health;
4. The first period with the newborn baby was a good time to make changes.

**A status report on my health and lifestyle—crucial for recognising the need for change**

Recognising the need for change was described as crucial for motivation to initiate change. Prior to the study, several participants had felt a need and desire to improve their lifestyle. For other participants, this realisation came during their participation in the study. The participants’ lifestyles and health were analysed through questionnaires and examinations involving blood tests, body composition, and blood pressure. For many, this provided better insight into their health and lifestyle. Dietary surveys were a revelation for many: ‘*It’s very interesting to see what you actually eat in the course of a week. It makes you feel a bit like, "Oh my God, is it really that bad?”*’ (Hilde). Similarly, body composition assessment gave the women a better understanding of their health and strengthened their motivation to make changes.

*You realise you’ve been on the wrong track. You get clear information about your state of health […] when you stand on those special scales and learn how much fat you’ve really got […] It’s not news to me, but it’s really good to get it kind of face to face, because I need a bit of a slap on my bottom.* (Frida)

In addition to seeing the results of the tests, the participants found it important and reassuring to have the significance of the results explained to them and be told how they could improve their measurements by making lifestyle changes. Learning about healthy dietary habits and how food affects the body increased their motivation to make healthy choices. Further, greater knowledge gave many of the women better insight into their eating behaviour.

*I eat till I’m full, but I don’t eat two more portions because I think it’s so delicious. Because I know I don’t need to […] it’s made me much more aware of food for myself and my baby. That’s like the biggest change for me, because I used to be a person who ate a lot more than I needed to be full. Just because it tasted good, as if you were never going to eat it again.* (Lea)

Participation in the study led the women to reflect on the care they had received for their GDM prior to the study, and on how much motivation to make lifestyle changes they would have had if they had not participated in the study. Several participants felt that they had received inadequate care and support. Some participants who were themselves healthcare professionals or interested in health and lifestyle described how they had to draw on their own background knowledge to compensate for inadequate support.

*Just having somebody to follow you up after you’ve had gestational diabetes, because I haven’t had that. You’re only recommended to contact your GP to have a long-term blood sugar test; that’s what you’re supposed to have once a year. Otherwise, that’s it.* (Hilde)

There was considerable variation among the participants in the postpartum follow-up they had received. The majority had had their long-term blood sugar levels checked by their doctor, but the information they had received about the connection between GDM, health risks and lifestyle varied greatly. Many felt that the focus was mostly on the management of GDM during pregnancy, but little on the postnatal period. The lack of information and follow-up was perceived by some as denying them the opportunity to improve their health. *‘I think it’s been really important to get that information* [about higher risk of disease after GDM], *because I’m young, so I have the chance to correct things if I want to.’* (Nina)

Other participants had been informed about their health risks and the importance of a healthy lifestyle, but this was insufficient to bring about change. They missed receiving support and guidance from healthcare professionals in the change process. They felt that the various clinicians had unclear roles, and several described being uncertain about whom to contact for support and advice.

*‘Eat healthy food or you’ll get diabetes’, that’s kind of the message you get, but you don’t hear much else. […] I haven’t thought about doing that either [asking my GP for information]. I was brought up to believe that you don’t go to the doctor unless you’re ill.* (Olea)

The way the information was presented by the professionals also affected how much insight they gained. Some participants described how clinicians had never talked to them about lifestyle or dared to tell them to lose weight. Many expressed a desire for direct and honest communication from healthcare professionals, where they were not shielded from reality. *‘I need to hear it. […] I don’t need to be mollycoddled’* (Frida). *’Doctors and nurses are in a unique position to be able to say: “I’m worried about your weight, and I’m worried about this and that”’* (Clara).

Different or contradictory information from healthcare professionals led to feelings of insecurity and confusion. For example, the seriousness of GDM was emphasised differently by different professionals, and there was great variation in recommendations for postpartum management. When asked what information she had received at the hospital for postnatal care of her GDM, Frida replied: *‘Nothing. I was told to just eat chocolate. Yes, that’s true. So that’s what I did’.*

**Encouragement and** **cheering on: getting started and maintaining changes**

Uncertainty about how to start the change process prevented several participants from initiating lifestyle changes. Some found it useful to set goals under the guidance of the study nutritionist. Keeping a record of their lifestyle and setting realistic goals motivated the women to change their habits. Individual advice was emphasised as more motivating than general advice. One of the women reflected on why she managed to change her lifestyle this time after several previous unsuccessful attempts. *‘Talking to someone who looks at my situation. Not general advice for everyone, but looking at my problem. […] Yes, that motivates me a lot more’* (Vårin). Sharing one’s goals with another person meant greater commitment. The regular phone calls with the nutritionist served as a reminder to stay focused on healthy lifestyle choices. The opportunity to share their change process with a supportive professional who offered praise and advice was an important boost for many participants:

*The conversations with the nutritionist really gave me a boost. I moved up a gear and got really enthusiastic, I felt like this was something important and possible. She was good at telling me not to overexert myself and try to do everything at once. They were very good conversations that sort of kept the iron hot. […] It’s been great to have someone who follows you up closely and gives you simple advice.’* (Lea)

Individual support enabled the participants to ask questions and receive answers from a reliable professional. This gave them a sense of security that motivated them to make positive lifestyle changes. The participants felt confident that they would make progress if they followed the lifestyle advice they were given. *‘If you follow the recommendations, you’re on the right track, but when you don’t follow them, you realise that you might not be able to achieve the goals you’ve set for yourself’* (Hilde). Exercise videos and information from the study website were important initial motivating factors. However, several participants called for more advanced exercises and new food recipes to keep up their motivation. Some also wanted more individual help than the study offered, such as a detailed, customised food and exercise plan.

The focus of the study on small lifestyle changes was a key factor in initiating the change process. Specific advice on bread and other food marked as healthy made it easier to implement the changes. Several participants expressed relief at being able to make lifestyle changes without turning their whole life upside down or going on a diet.

*‘What I liked was the less strict approach. You don’t have to change your whole life and go on a diet and all that, but there are lots of small steps that help with the big picture. […] We’ve made a lot of small changes that aren’t really that difficult, but they’re still important.’* (Else)

Some of the participants had an ‘all or nothing’ attitude, where they had previously thought that lifestyle change had to be perfect to be of value. This had often meant giving up trying to change if they made one unhealthy choice. The focus on small changes helped many to realise that the key to long-term success lay in finding a healthy lifestyle that worked in everyday life. *‘You just have to keep focused. Even if you occasionally make stupid choices, you can get back on track and accept your mistake: “OK, that wasn’t very healthy food now, was it, but we can make up for it next time”’* (Else). Reducing the demands on oneself increased the feeling of satisfaction and mastery. Several of the women found it motivating to realise how big an effect small changes had on their physical fitness and energy levels. Further, improvements in body composition, weight, blood pressure, and blood tests provided a sense of mastery and new motivation to make more changes. *‘I’ve kept all those sheets of paper* [of my results], *haha, it’s so much fun to see my progress. It’s motivated me a lot to see that things are improving’* (Nina).

Just as support from professionals motivated the participants, it was also important to be ‘cheered on’ by their partners and family members. It was easier to make lifestyle changes when partners and other family members were supportive, open to change, and cooperated to create time and space for healthy choices. Here, crucial aspects were practical help such as cooking and looking after the baby, as well as the feeling of working towards a common goal. The women’s motivation to maintain healthy habits was greater when it had positive effects on the whole family. ‘*I think my partner tries to eat more vegetables when he sees me doing it. And as I want wholemeal bread, he and the kids have to eat it too, haha*’ (Amalie). However, some participants only received passive support from their partner and family. They were in favour of the woman’s change but were not involved in the change process themselves. This resulted in a feeling of having sole responsibility for the lifestyle changes. One participant described how she had to cook brown rice for herself and white rice for the rest of her family. When asked how this affected her, she replied: *‘It was much easier to slip up if it was his turn to make dinner, I didn’t always have the energy to cook something else’* (Pia). Participants agreed that interest and involvement by their partner had a positive influence on their lifestyle changes. Many therefore suggested more active inclusion of partners in any future intervention study.

*I think that could be a resource because then you’re kind of both in it together. Because you don’t normally have dinner alone, do you? If everyone in the house eats healthy food, it automatically has a positive effect. (Torill)*

It was also emphasised that it was more motivating to make lifestyle changes with others. Several participants missed being in a group. Exercise groups and social get-togethers with others in the same situation were highlighted as factors that facilitated lifestyle changes. There was a suggestion to have a digital group where the women could ask questions, share knowledge, and be inspired by one other. Comments from others who were proud of the changes they had made or who asked for tips on lifestyle changes provided a sense of achievement and motivation to maintain the changes.

**Life’s challenging moments:** **looking after the baby and prioritising one’s own health**

Although the participants wanted to make lifestyle changes, there were many barriers. Many felt overwhelmed in the first year of their child’s life. They needed time to adjust to parenthood and their new life and found it difficult to think about their own health and lifestyle at the same time. *‘The postnatal period is tough. You get tired a lot, lots of hormones, lots of things going on, so I know it’s easy to resort to sweet things or not take any exercise because you feel generally worn out’* (Gina).

The needs of the participants’ children outweighed the need to make good choices for their own health in several cases. One participant described spending a great deal of time and effort ensuring a healthy diet for her children while her own diet had lower priority. Several had a guilty conscience for their children if they decided to spend time on themselves. This often made them avoid exercising. However, it was easier to exercise if the children also enjoyed the activity.

*More programmes where you can bring your children would be really great. Because that’s what I’m struggling with, I can’t quite manage to prioritise myself. But if it’s something I can see my little boy will also enjoy, it’s that much easier to get involved. […] it’s also about convincing yourself, thinking that you’re doing it for your child and not necessarily just for yourself.* (Mona)

The loss of everyday routines due to holidays and various celebrations made it difficult to maintain lifestyle changes. Such occasions often involved more unhealthy food and less activity, and increased the need to relax and enjoy life. Several participants found it challenging to deal with other people’s expectations to celebrate in the traditional way by stuffing oneself with unhealthy food.

*Now at Christmas time, there’s a lot of talk about food and sweet things and all that. There are so many temptations on offer, but I just try to stick to what I usually eat. It’s a bit boring, and I feel I’m a bit boring too. […] There’s some pressure, you know: ‘What - you don’t eat this, and you don’t eat that?’.* (Janne)

The transition from maternity leave to work was described as particularly demanding. Several participants found it difficult to maintain the lifestyle changes from their maternity leave when they went back to work again. They felt they had to start all over again and plan differently to make sure they still had healthy habits.

*Maybe that’s what dragged me down, being suddenly faced with really busy days again. And that’s what makes you resort to easy solutions’ (Kari). ‘It would have helped if I’d had an extra phone call [from a nutritionist] during the changeover period, because that was a tough time for me. […] When you’ve got a baby on top of everything else, you have to plan things a bit more.’* (Nina)

The period following the birth was busy, with little sleep and energy. It was difficult to maintain a focus on lifestyle in such a demanding life situation, which could include disruptive changes such as relationship problems or changing jobs. Some participants described how such situations could lead to a relapse into old habits. Several participants called for advice that would increase their chances of keeping to their newly established lifestyle changes despite these important barriers. *‘Stress during busy periods, maybe that’s my biggest challenge. […] If I’m very stressed and very busy, it’s easy just to grab a chocolate or something, just something simple, easy and quick’* (Else).

**The first period with the newborn baby—a good time to make changes**

The maternity leave period was considered by many participants to be a suitable time for lifestyle change because it gave them more time and flexibility. *‘Now I had a new baby in my arms, I was at home during the day. That was a good opportunity to try and fix a diet that maybe wasn’t as good as it ought to be’* (Nina). When asked about the best time to start a lifestyle intervention program after GDM, most women stated that 3–6 months postpartum—after establishing the first routines with their newborn but before returning to work—was the most suitable time. Although the participants described many barriers to maintaining lifestyle changes, they were all motivated by the arrival of the newborn baby in their lives. The baby’s best interests were a key motivational factor for lifestyle change for most participants. While the baby was still in the womb, this motivated them to have a healthier lifestyle because their choices directly affected the baby. Some found it difficult to maintain the healthy changes after giving birth, but participation in the study was a golden opportunity to continue.

*I thought it [joining the study] was an excellent opportunity not to let go completely after giving birth. You know, when you have gestational diabetes, it’s like you just wait until you’ve given birth, so you can eat almost anything you want again without worrying about your blood sugar.* (Amalie)

A strong desire to be a good role model for one’s children was a recurring theme. Several were also motivated by the desire to have the energy to participate in their children’s activities. *‘You don’t think about what’s going to happen in ten years’ time. Now I’ve become more aware of how I want to be and feel in the future. I want my child to have a mum who can join in family activities properly and isn’t just a hanger-on’* (Lea).

Some women were simply motivated by the need to prioritise themselves after the pregnancy. One of them stated: ‘When your pregnancy and labour are over, it feels great and reassuring to get that extra motivation and help to start taking your body back’ (Nina). Others focused more on the sense of well-being that came with a healthy lifestyle, with more energy, less pain and a lighter feeling. When asked what motivated her, Kari replied: ‘The experience of good health. Feeling that you’re fit and you’ve been moving your body, the feeling you get after exercise’. Better mental health was also mentioned as a motivating factor. The desire to prevent GDM in the next pregnancy and reduce the increased risk of cardiovascular disease associated with a GDM diagnosis encouraged the participants to change to more healthy habits. Having family members with lifestyle diseases reminded them of the importance of positive change.

*We have heart problems in our family on my father’s side, so it’s not unrealistic that something could happen to me too. […] So it was a bit of an eye-opener when we started to get signs that things weren’t quite right. It makes you pull yourself together, you realise you can’t just wait till your children have moved out.* (Mona)

## 4. Discussion

This qualitative study explored participants’ experiences of postpartum follow-up and participation in a lifestyle intervention programme following GDM. The study revealed that sufficient information and insight into one’s health, specific advice, and a focus on small changes were key motivational factors for initiating lifestyle changes. Factors helping to maintain change were support from professionals and one’s social network in the change process, noticing an improvement, and seeing a positive effect on family members. Maternity leave was emphasised as a good time for change. However, the participants had difficulty in maintaining healthy habits when they started work again and in challenging life situations. Other barriers to lifestyle change were limited time and energy, disruptions to everyday routines, and prioritisation of their children over themselves.

Previous studies have shown that knowledge of a future risk of diabetes and of the importance of a healthy lifestyle are key factors in recognising the need for and implementing lifestyle change [17,18]. Similarly, our participants stated that the information in the study about GDM, health risks, and lifestyle increased their understanding of their health and motivated them to improve it. In addition to greater knowledge, participants in our study described how measurements and dietary surveys in the intervention increased their insight into their health.

Our findings of the perceived inadequacy of postpartum follow-up in the healthcare system are supported by a number of studies that have explored postpartum follow-up after GDM [10,13,19]. National guidelines for postpartum care of women with GDM in Norway recommend regular monitoring of HbA1c, information about increased disease risk, and lifestyle advice, as well as practical support for lifestyle change [1]. The majority of our participants reported having measured their HbA1c level postpartum, but they received varying information about increased risk of diabetes, cardiovascular disease, and lifestyle changes. This concurs with findings from a recent Norwegian study showing that few of the participants received follow-up care as stated in the guidelines, while they experienced poor support from primary healthcare [10,11]. A Danish study found that several women with GDM felt that there was little initiative for further care and support from the healthcare system to achieve lifestyle changes postpartum [10], despite the fact that women’s need for professional support to succeed has been shown in several studies [13,20,21].

As seen in the findings of an American intervention study of women with GDM [22], our study found that the intervention programme with individual support from a nutritionist was considered important for successful lifestyle change. The nutritionist’s support provided individualisation and improved accountability. An Australian study that investigated the factors promoting and hindering physical activity postpartum concluded that individualised exercise programmes could increase activity levels. This was because the women varied in their preferences for type of activity and in their barriers to being active [23]. Several participants in our study also called for more personalised diet and exercise plans, as in the findings from the Australian study.

As in many other studies, our participants described a lack of time and energy as barriers to a healthy lifestyle [10,18,23]. Advice and guidance on how to ensure healthy habits in a busy life was commonly mentioned in the interviews. Several participants found that the intervention programme’s focus on small changes based on a normal diet, instead of a special diet, made lifestyle change more manageable. This is in line with recommendations in a systematic review [24].

As in the present study, previous studies have found that diabetes prevention programmes should take into account women’s multiple roles as carers, workers, and patients to increase the likelihood of long-term lifestyle change [25]. A focus on changes that benefit the whole family has also been found to improve adherence [25]. Several participants in our study described how they prioritised their family’s needs over their own health. The need for baby-sitting is therefore mentioned as a natural barrier to being physically active in the present and previous studies [23,26]. Drawing on this knowledge, our intervention study emphasised flexibility, stating that a baby-sitting arrangement was not a requirement for participation [27].

The desire to be good role models and to stay healthy for the sake of their children was expressed by several participants, which is also commonly found in other studies [18]. However, some participants in our study had difficulty in maintaining lifestyle changes after giving birth because their lifestyle no longer affected the child directly, which is in line with findings from a Danish study [10].

Other studies agree with our findings that crucial factors for successful lifestyle change are support from partners, family members, friends, or other social networks such as forums or group exercise sessions [18]. Of particular importance is the role of the woman’s partner in facilitating lifestyle changes and being involved and supportive in the process [11,26]. Similar to other studies, several women in our study expressed a desire for greater partner involvement in their lifestyle changes [10,18]. Studies that have included partners in intervention programmes have found that both the partner and the woman felt that the involvement was motivating and useful [28].

The transition from maternity leave to work was described as challenging, and many women called for more help to maintain healthy habits during this phase. Further, changes and disruptions to normal everyday life were pointed out as barriers to lifestyle change. In line with this, a systematic review recommends offering women additional support in demanding life situations that may prevent them from maintaining a healthy lifestyle [24].

The start of our intervention programme varied between three and twelve months postpartum, and all participants felt that maternity leave was a good period for lifestyle changes due to more time and flexibility. An American study found that women felt ready to initiate lifestyle changes six weeks postpartum [22], while a Danish article showed that 3–6 months after birth was appropriate [18]. Differences in maternity leave schemes in different countries are likely to affect what women perceive as an appropriate time to start lifestyle interventions.

The study population consisted of women who agreed to participate and complete a lifestyle intervention. They may represent a group that is more motivated to make lifestyle changes, and their experiences are therefore not necessarily representative of the general population of women in Norway with GDM.

However, the participants varied in age, marital status, educational level, household income, family history of diabetes, BMI, and parity. The broad range of key characteristics that may have influenced the participants’ experiences with their postpartum follow-up and the intervention programme ensured representation of important aspects of the research topic. However, only Norwegian-speaking women were included and only two of the participants were born in countries outside Scandinavia. The prevalence of GDM in Norway is higher in immigrants from Asia and North Africa than in non-immigrants [29]. This is thus a limitation as these groups may have experiences and challenges that are not represented in our study.

The research team involved in this study are all healthcare professionals with great interest in health and lifestyle. The team may have had blind spots that affected the interpretation of the data, while a broader research team might have mitigated this risk. However, continuous reflection and the inductive approach in the analysis helped to increase the objectivity of the findings. Telephone interviews prevented the interviewer from interpreting the participants’ body language, which may have affected the understanding of their feelings and experiences. On the other hand, telephone interviews enabled us to reach a greater number of participants. In addition, participants may have found it easier to give their honest opinion about the study over the phone than face-to-face. Our findings regarding participants’ need for adequate information, individualised help, support from others, and additional support in demanding life situations, align with similar studies from more diverse populations from high-income countries [24]. However, participation in the intervention programme required access to the internet and capability of communicating in Norwegian and the study’s findings may not be directly applicable to other contexts. It is also important to keep in mind that the study was conducted in a country with universal healthcare access, parental benefits, and national screening recommendations for GDM. Poor finances were rarely mentioned as a barrier to a healthy lifestyle. This may limit the transferability to other countries without free healthcare, where more of this group of women may have everyday financial challenges. In addition, there is a longer period of parental leave in Norway than in many other countries, and the time to start the intervention preferred by our participants is not necessarily transferable to countries with other leave schemes.

## 5. Conclusions

Participants in the MHH intervention programme emphasised mid-maternity leave as a good time for initiating lifestyle changes. They underlined the need for adequate information, professional guidance, and support from family and friends to be essential for successful lifestyle change. Individualised support, a focus on small changes, specific advice, and clinical measurements increase motivation for lifestyle change and should be included in future interventions. Further, later interventions should involve partners to a greater extent and offer closer follow-up in challenging phases such as the transition between maternity leave and work.

## Figures and Tables

**Table 1:** Participant demographics and pregnancy characteristics (n = 22).

Characteristic		n (%)
Age (years)		
	<30	5 (23)
	30–34	9 (41)
	≥35	8 (36)
Ethnicity		
	Norwegian	20 (91)
	Other	2 (9)
Marital status		
	Married	7 (32)
	Cohabiting	15 (68)
Education		
	Secondary education	3 (14)
	Lower tertiary education (<4 years)	6 (27)
	Upper tertiary education (≥4 years)	13 (59)
Household income		
	NOK <750,000	6 (27)
	NOK 750,000–1,000,000	5 (23)
	NOK >1,000,000	10 (45)
	Missing	1 (5)
Parity		
	Primiparous	6 (27)
	Multiparous	16 (73)
Gestational age		
	<37 weeks	3 (14)
	≥37 weeks	19 (86)
Time since delivery (at time of the interview)		
	9–11 months	10 (46)
	12–14 months	8 (36)
	15–18 months	4 (18)
Family history of diabetes		
	Yes	5 (23)
Smoking *		
	Never	16 (73)
	Former	6 (27)
	Current	0 (0)
Body mass index *		
	<25 kg/m^2^	4 (18)
	25–<30 kg/m^2^	12 (55)
	≥30 kg/m^2^	6 (27)

* At baseline after recruitment in MHH.

## Data Availability

The interviews transcribed for the present study are not publicly available due to individual privacy considerations. All data requests should be submitted to the corresponding author for consideration. Access to anonymised data may be granted upon reasonable request, subject to approval by the Central Norway Regional Committee for Medical and Health Research Ethics.

## Supplementary Materials

The following supporting information can be downloaded at: https://www.mdpi.com/article/10.3390/nu16203487/s1, Table S1: Interview guide. Figure S1: Thematic map.
