# Supplementary material for: Experiences of Postpartum Follow-Up and Participation in a Lifestyle Intervention after Gestational Diabetes: A Qualitative Study
Source: Nutrients. 2024 Oct 15;16(20):3487. doi: 10.3390/nu16203487 (PMC11510314; doi:10.3390/nu16203487)
Supplement: Supplementary file 1 [file nutrients-16-03487-s001.zip › Figure S1 Thematic map.pdf]

Figure S1. Thematic maps developed during data analysis.

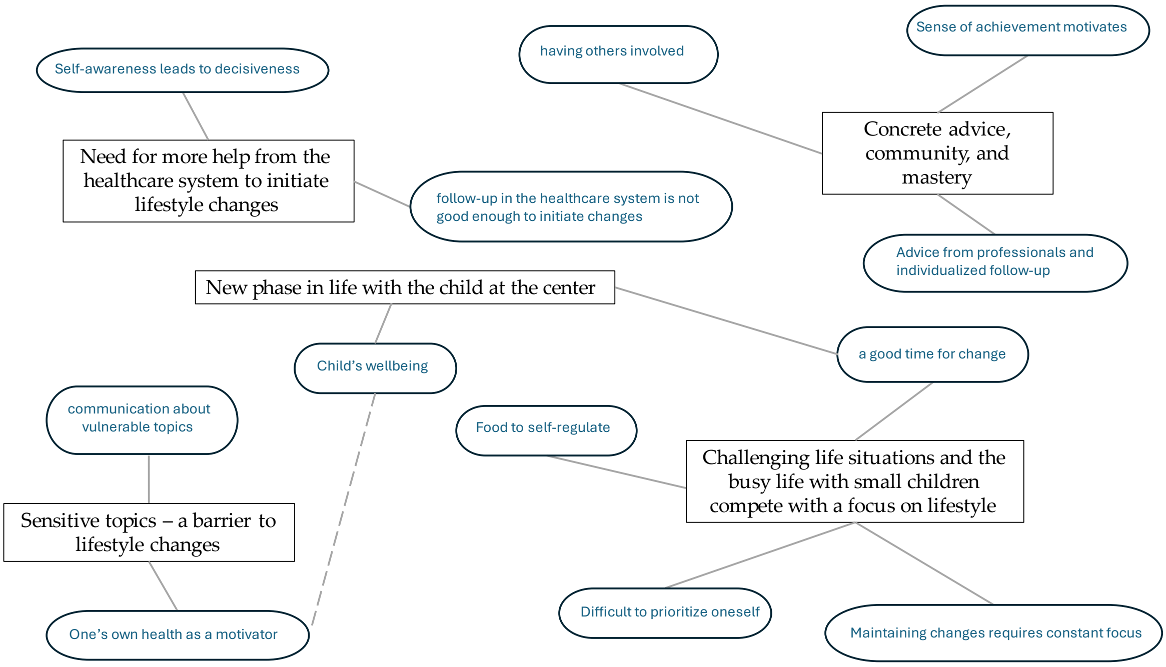

Figure S1a. Initial thematic map.

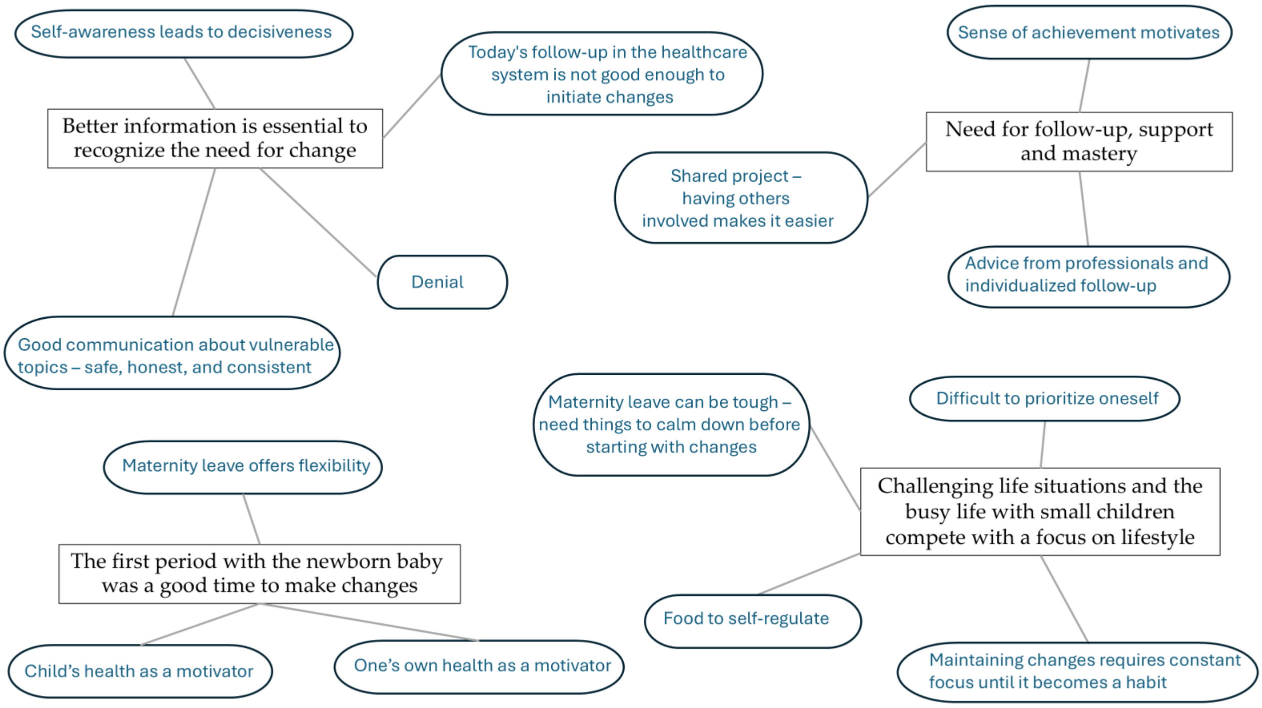

Figure S1b. Developed thematic map.

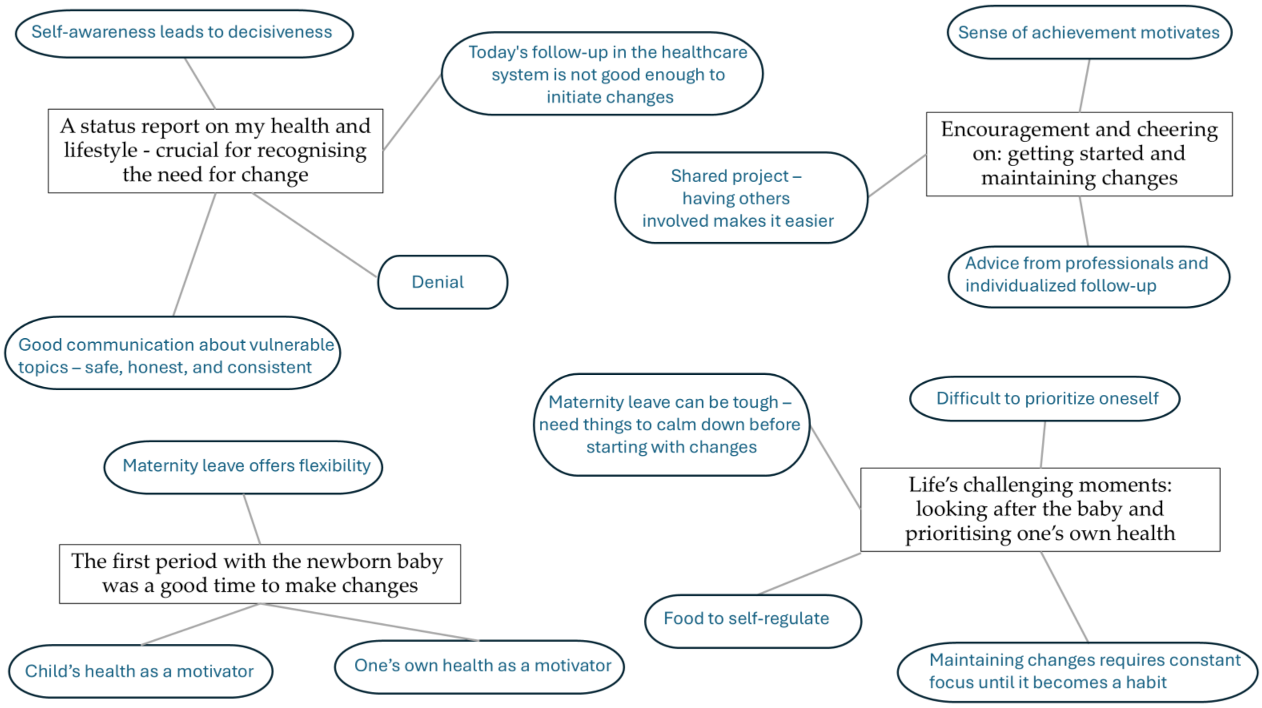

Figure S1c. Final thematic map
