# Supplementary material for: Experiences of Postpartum Follow-Up and Participation in a Lifestyle Intervention after Gestational Diabetes: A Qualitative Study
Source: Nutrients. 2024 Oct 15;16(20):3487. doi: 10.3390/nu16203487 (PMC11510314; doi:10.3390/nu16203487)
Supplement: Supplementary file 1 [file nutrients-16-03487-s001.zip › Table S1 Interview guide.pdf]

Table S1: Interview guide.

Thank you for taking part in our “Mom’s Healthy Heart” study. We are interested in hearing about your experience of the study. We would also like to hear what support you have received from health care providers since the birth and during the study.

Experiences of the intervention/study

Could you please describe what you thought when you received the letter of invitation to participate in the study?
What motivated you to take part?

What are your experiences of taking part in the study? What did you like about the study?
What didn’t work very well/didn’t work at all?
Was there anything you missed? If so, what did you miss?

Please give us some suggestions on how we can improve the study.

What did you think of:
- The content and user-friendliness of the website?
- The personal advice and guidance?
- The number of sessions and time between sessions?
- The physical examinations and questionnaire?
- The use of time?
- Feasibility?
- Recruitment/timing?

Has participation in the study benefited you personally? If so, in what way?

Have you changed anything in your lifestyle during the study?
If so, what?
Do you think that you will continue the lifestyle changes on an everyday basis in the future?
If yes:
- What might encourage/help you to continue the changes?
- What obstacles might you face?

Has your participation in the study had an effect on the lifestyle of the rest of your family?
If yes, how/what, which family member(s)?

Have people around you supported you during the study?

If yes, who and how?

If no, why not?

How have things been at work? What is your job?

Do you have any suggestions for how people around you could have given you better support?

Do you think that some groups might be difficult to recruit to the study? If so, why?

Experience of follow-up care after the birth

Please describe the follow-up care you received from health care providers after the birth:

- In the maternity ward
- From your primary care physician

Do you feel that the follow-up care after the birth was adapted to the complications you had during your pregnancy?

What did you expect or want from your primary care physician and the maternity ward?

Knowledge of the link between preeclampsia/gestational diabetes and future health

What did you know about preeclampsia/gestational diabetes and the risk of cardiovascular disease before you took part in this study?

How did you get information about follow-up care after the birth? What information did you get? From whom?

How do you feel about the way health care professionals have spoken to you about this topic?

Examination/check-up after the birth

Have you heard about a screening examination/health check-up after the birth to detect high blood pressure or diabetes?

Have you had an examination/check-up to detect high blood pressure or type 2 diabetes? What was your experience of this?

How did you find out about the examination/check-up? What made you attend/not attend at that point?

Is there anything that could have made it easier to attend that kind of check-up? If so, what?

Do you think health care professionals should encourage women to have a health check-up? Why?

What do you think is the advantage of having the check-up?

What do you think are the disadvantages of having the check-up?

Who do you think is responsible for carrying out this check-up? Who do you expect help from in connection with the check-up?

Preventive treatment/lifestyle changes

Have health care professionals (apart from Mom's Healthy Heart) ever discussed preventive treatment or lifestyle changes with you (diet, physical activity, body weight or smoking)?

If yes, who and when?

About what?

What did you think of that discussion? How important is that kind of discussion, in your opinion?

Do you want to receive support and advice from health care professionals?

If yes, from whom?

What support or advice?

Did you get advice or guidance from health care professionals (apart from Mom's Healthy Heart) during this study?

How important was this support/lack of support to you?

Collaboration and communication with/between health care professionals

How do health care professionals react to your questions?

Please describe the collaboration/communication between the different health care professionals you have contact with?

Final question:

Is there anything you would like to ask or add?
